# Heads, I Win, Tails You Lose

**Published:** 1897-09

**Authors:** 


Heads, I Win, Tails You Lose.—Our distinguished contemporary of the *Texas Medical News* argues, and quite correctly, that a physician of experience, backed by a theoretical knowledge of disease acquired at college, is better qualified to teach medicine than a young man without practical experience, however good his theoretical knowledge. And yet he would turn out the yellow fever veterans in charge of State quarantine, and trust the protection of the people from yellow fever to the young men of the Marine Hospital Service who have never seen a case of the fever. Young Branan's fate alone, should convince our neighbor that experienced physicians are better able to deal with yellow fever than are novices, as well as better qualified to teach medicine.

